# Regulatory mechanism of ghrelin on testosterone secretion in type 1 diabetic rats

**DOI:** 10.1530/RAF-24-0087

**Published:** 2025-07-21

**Authors:** Chien-Chen Lu, Chia-Hsin Chang, Jou-Chun Chou, Po-Ling Yu, Paulus S Wang

**Affiliations:** ^1^ Department of Optometry, MacKay Junior College of Medicine, Nursing, and Management, Taipei, Taiwan; ^2^ Department of Physiology, School of Medicine, National Yang Ming Chiao Tung University, Taipei, Taiwan; ^3^ Department of Medical Research, Shuang Ho Hospital, Taipei Medical University, New Taipei, Taiwan; ^4^ Continew Medical, INC., Taipei, Taiwan; ^5^ Medical Center of Aging Research, China Medical University Hospital, Taichung, Taiwan; ^6^ Department of Biotechnology, College of Health Science, Asia University, Taichung, Taiwan

**Keywords:** rats, STZ, LH, LHR, TE

## Abstract

**Abstract:**

Ghrelin, which is a hormone composed of 28 amino acids that is mainly produced in the stomach, is also secreted by Leydig cells in the testes of rats and humans. The hypothalamus regulates testosterone secretion by releasing gonadotropin-releasing hormone (GnRH), which stimulates the pituitary gland to release luteinizing hormone (LH). LH then prompts the testes to produce testosterone via the activity of steroidogenic acute regulatory protein (StAR). Consequently, ghrelin may play a regulatory role in gonadal function. Male Sprague–Dawley rats were randomly assigned to four groups: the control, ghrelin-treated, diabetic, and diabetic plus ghrelin treatment groups. After the rats were sacrificed, plasma samples were collected. Leydig cells were isolated and cultured with human chorionic gonadotropin (hCG, which is similar to LH and is used to stimulate Leydig cells to synthesize testosterone), 8-bromoadenosine 3′,5′-cyclic monophosphate (8-Br-cAMP, which is an activator of cyclic adenosine monophosphate-dependent protein kinase), or forskolin (an activator of adenylyl cyclase in a wide variety of cell types). Compared with normal treatment, ghrelin treatment in diabetic rats markedly increased plasma testosterone levels by 3.75-fold (*P* < 0.05), Leydig cell testosterone secretion by 2.8-fold (*P* < 0.05), GnRH-mediated LH release from the anterior pituitary by 2.95-fold (*P* < 0.05), and StAR expression by 1.96-fold (*P* < 0.05) in testicular Leydig cells. These findings indicated that ghrelin enhanced testosterone production in diabetic rats, which was partially achieved by the hypothalamic–pituitary–gonadal axis and StAR. This study emphasized the potential use of ghrelin as a treatment for improving testosterone levels and gonadal function in individuals with diabetes.

**Lay summary:**

This study explored how a hormone known as ghrelin (which is mainly produced in the stomach) may help in regulating testosterone levels. Researchers examined male rats in four groups, including diabetic rats treated with ghrelin, and discovered that ghrelin increased testosterone production in diabetic rats by improving the communication between the brain, pituitary gland, and testes. This hormone also helped specific cells in the testes to function more effectively. The diabetic rats treated with ghrelin exhibited notable increases in testosterone levels and improved hormone function. These findings suggest that ghrelin could potentially help to address hormonal imbalances related to diabetes and improve reproductive health. This research highlights the potential benefits of ghrelin in addressing hormonal imbalances.

## Introduction

Diabetes mellitus (DM) significantly disrupts various physiological systems, including reproductive function (Antar *et al.* 2023). Sexual dysfunction has been reported in 30–70% of male diabetic patients, thereby underscoring its prevalence as a major complication of diabetes (Corona *et al.* 2013, Bahar *et al.* 2020). Experimental studies have revealed that diabetes impairs testicular function, as evidenced by decreased testicular weight, reduced Leydig cell count, lower testosterone levels, and diminished sperm volume in diabetic rats (Ding *et al.* 2015, Sayed *et al.* 2023). These impairments stem from chronic hyperglycemia, which disrupts the hypothalamic–pituitary–gonadal (HPG) axis and compromises gonadotropin activity, particularly regarding luteinizing hormone (LH) secretion (Iliff-Sizemore *et al.* 1990, Costanzo *et al.* 2014).

The HPG axis plays a critical role in testosterone synthesis (Dufau 1998), and hormonal imbalances play a crucial role in diabetes-induced reproductive dysfunction. LH, which is a primary regulator of testosterone synthesis, binds to luteinizing hormone receptors (LHRs) on Leydig cells, thereby activating signaling cascades mediated by cAMP and protein kinase A (PKA) to increase steroid hormone production (Neuman *et al.* 1991, Dufau 1998, Mendez *et al.* 2003). Chronic hyperglycemia disrupts gonadotropin-releasing hormone (GnRH) secretion and subsequent gonadotropin activity, thus exacerbating reproductive dysfunction in diabetic patients (Iliff-Sizemore *et al.* 1990). In addition, the LHR binds human chorionic gonadotropin (hCG), which shares structural and functional similarities with LH and plays a significant role in testosterone regulation (Choi & Smitz 2014, Casarini *et al.* 2018). In diabetes, chronic hyperglycemia suppresses GnRH secretion, thus reducing LH levels and contributing to hypogonadism (Dhindsa *et al.* 2004). Recent studies have confirmed that diabetes consistently reduces testosterone levels and Leydig cell function, thereby underscoring the need for targeted interventions (Ding *et al.* 2015, Huang *et al.* 2024).

Energy metabolism-related peptides, such as leptin and ghrelin, have garnered attention for their roles in regulating both metabolic and reproductive functions (Fernandez-Fernandez *et al.* 2006). Ghrelin, which is a 28-amino-acid peptide discovered in 1999, stimulates appetite, regulates energy homeostasis, and modulates growth hormone secretion via the growth hormone secretagogue receptor (GHS-R) (Date *et al.* 2000, De Vriese & Delporte 2008, Alamri *et al.* 2016). Ghrelin levels are inversely correlated with body mass index (BMI) in healthy individuals, thus further indicating its role in energy metabolism (Alamri *et al.* 2016). In addition to these metabolic functions, ghrelin and its receptors have been identified in reproductive tissues, including the testes and ovaries, thereby suggesting its involvement in modulating the HPG axis (Rindi *et al.* 2004, Greenman *et al.* 2009).

Previous studies have indicated that ghrelin influences gonadal function by regulating the hypothalamic–pituitary axis and directly affecting gonadal tissues (Tena-Sempere *et al.* 2002, Fang *et al.* 2012). In diabetic conditions (wherein energy metabolism and hormonal imbalances exacerbate hypogonadism), ghrelin may offer therapeutic potential.

This study investigated the role of ghrelin in regulating gonadal function and enhancing testosterone synthesis in a rat model of type 1 diabetes. We hypothesized that ghrelin administration could counteract diabetes-induced suppression of testosterone production by stimulating LH release at the anterior pituitary (AP) and enhancing steroidogenic capacity in the testes. By elucidating the effects of ghrelin on reproductive dysfunction, this research aimed to provide valuable insights into effective therapeutic strategies for managing hypogonadism associated with diabetes.

## Materials and methods

### Experimental animals

Sprague–Dawley (SD) male rats were procured from the Yang Ming Chiao Tung University Animal Center. The rats were housed in a controlled environment under a 14 h light:10 h darkness cycle (lights on at 06:00 h and off at–20:00 h), at 22 ± 1°C, with access to food and water *ad libitum*. All of the experimental protocols adhered to the Guidelines for the Care and Use of Laboratory Animals (8th edition) and were approved by the Institutional Animal Care and Use Committee (IACUC, National Yang Ming Chiao Tung University, Taipei, Taiwan, permit number: 1040409). The determinations of the sample size and the number of animals to be used in the study were based on prior studies (Tsai *et al.* 2003*a*,*b*), recommendations from Charan & Kantharia (2013), and recommendations from the Animal Research: Reporting of *in vivo* Experiments (ARRIVE) guidelines (Percie du Sert *et al.* 2020).

### Type 1 diabetes rat model

Type 1 diabetes was induced using the method described by Mansford & Opie (1968). Streptozotocin (STZ), which is a natural alkylating agent that is toxic to pancreatic beta cells, was utilized in this study. The structural similarity of STZ to glucose allows for its uptake via GLUT2 glucose transporters, thereby leading to selective beta-cell death and hyperglycemia (Ledoux & Wilson 1984, Elsner *et al.* 2000). Male SD rats were fasted for 24 h before injection with 50 mg/kg STZ dissolved in 0.1 M citrate buffer via the tail vein (Lin *et al.* 1991). On the third day post-injection, blood glucose levels were measured using an ACCU-CHEK Advantage blood glucose meter (Roche, USA). Rats with glucose levels exceeding 200 mg/dL were classified as diabetic and included in the subsequent experiments. The control group (vehicle group) received an equivalent volume of citrate buffer.

### *In vivo* experiments

#### Effect of ghrelin on plasma testosterone in male rats

The rats were randomly allocated into the following four groups.

- **Control group:** no treatment.
- **Ghrelin-treated group:** rats received daily tail vein injections of ghrelin (10 μg/kg) for 7 days.
- **Diabetic group:** rats were pretreated with STZ (50 mg/kg, i.v.) to induce type 1 diabetes.
- **Diabetic-ghrelin group:** diabetic rats received daily ghrelin injections (10 μg/kg) for 7 days.

The dosages and injection duration were selected based on previous studies of ghrelin (Masuda *et al.* 2000, Liu *et al.* 2016). After the rats were sacrificed, blood samples were collected, and the plasma was separated. Ether extraction was performed, followed by air drying and reconstitution in 0.1% gelatin-PBS. Testosterone concentrations were analyzed using radioimmunoassay.

### *In vitro* experiments

#### Preparation of Leydig cells

Testes were collected, and Leydig cells were isolated using the Percoll gradient method (Wang *et al.* 1999, Chiao *et al.* 2002). Decapsulated testicular tissue underwent collagenase dispersion and preincubation in medium containing 1% BSA, HBSS, and HEPES. After aeration with 95% O_2_ and 5% CO_2_ and incubation at 34°C in a shaking water bath, the digested cells were filtered and centrifuged. Red blood cells were disrupted using hypotonic shock. The cell suspension was prepared in medium 199 with 2.2 g/L sodium bicarbonate at a concentration of 1.0 × 10^6^ cells/mL, with over 97% viability being achieved. The Leydig cell abundance, which was quantified using 3β-HSD staining, was observed to be approximately 18 ± 2%. The protein content was measured by the Lowry method.

#### Effect of ghrelin pretreatment on testosterone synthesis

Leydig cells were cultured in medium lacking phenol red to avoid interference with testosterone secretion (Welshons *et al.* 1988). To analyze testosterone synthesis, the cells were exposed to the following treatments:

- Vehicle control.
- hCG (0.05 IU/mL).
- 8-Br-cAMP (a cAMP analog, 10^−4^ M).
- Forskolin (an adenylyl cyclase activator, 10^−5^ M).

After one hour of incubation, the reaction was halted with ice-cold 0.1% gelatin-PBS. The samples were centrifuged to collect the supernatant for radioimmunoassay or stored at −20°C for further analysis.

#### Effect of ghrelin on LHR and StAR expression

Leydig cells (2 × 10^6^ cells per group) were cultured for one hour. Following centrifugation, the cell precipitates were subjected to Western blotting analysis for the detection of LHR and StAR protein expression, whereas the supernatant was subjected to radioimmunoassay to measure testosterone secretion.

### Radioimmunoassay for testosterone

Radioimmunoassay protocols that were previously established in our laboratory were used to measure testosterone concentrations in the Leydig cell culture medium and plasma (Tsai *et al.* 1996). The sensitivity of detection was 2 pg/tube, with intra-assay and inter-assay variation coefficients of 4.1 and 4.7%, respectively. Antibody cross-reactivity with steroid substances was minimal (Wang *et al.* 1994).

### Anterior pituitary (AP) culture

The *in vitro* culture of AP tissues has been previously established in our laboratory (Tsai *et al.* 2003*a*). The AP gland was removed and bisected. The cells were stabilized by basal culture for 1.5 h. Each hemi-AP from the same rat was subsequently placed in a culture flask containing 1 mL of medium (either without (control group) or with 10 nM GnRH) and incubated for 1 h. Following incubation, the tubes were placed on ice to halt the secretion of LH. The AP was subsequently removed and weighed, and the culture medium was collected. The LH concentration in the medium was measured using a radioimmunoassay, or the supernatant was stored at −20°C for subsequent LH analysis. LH levels were normalized to the weight of the corresponding hemi-AP sample.

### Radioimmunoassay for LH

The LH concentrations in the AP culture medium were measured using established protocols (Wang *et al.* 1994). The LH concentration in the samples was calculated using the log-logit transformation method. In general, the maximum binding rate of the antibodies was greater than 30%, the sensitivity was 47 pg/tube, and the intra-assay and inter-assay coefficients of variation were 2 and 4.5%, respectively.

### Western blotting analysis

The specific Western blotting method utilized in this study has been previously described (Tsai *et al.* 2003*b*, Yu *et al.* 2009, Lu 2021). The soluble proteins in the clarified lysates were quantified by the Bradford method (Bio-Rad protein assay, Bio-Rad Laboratories, Inc., USA), and bovine serum albumin (BSA, Sigma-Aldrich, USA) was used as the standard. The whole-cell lysate proteins were detected in 50 μg aliquots of each protein sample, resolved via SDS-PAGE (10% acrylamide), transferred onto PVDF membranes (NEN Life Science Products, USA), and immunoblotted with specific antibodies.

The primary antibodies against the LHR (Santa Cruz Biotechnology, USA) and StAR (provided by Dr D M Stocco, Health Science Center, Texas Tech University, USA) were used in this study, with alpha-tubulin (Sigma-Aldrich) being used as a loading control (Chen *et al.* 2017, Lin *et al.* 2020). Horseradish peroxidase-conjugated rabbit, mouse, or goat secondary antibodies (Jackson ImmunoResearch Laboratories, USA) were used with a chemiluminescent detection system, as previously described (LAS-4000 mini, Fujifilm, Japan).

### Statistical analysis

All of the data are presented as the means ± standard errors of the means (SEMs). The data for each experimental condition were analyzed using one-way analysis of variance (ANOVA) to assess differences among the treatment groups, as the study design focused on a single factor (treatment). This approach was chosen over the use of a two-way ANOVA because only one independent variable was evaluated in each analysis. When significant differences were detected, Duncan’s multiple range test was applied for post hoc analysis to identify specific differences between the group means (Steel & Torrie 1980). No data were missing in this study, and no imputation was performed. For the *in vivo* experiments, a sample size of 5–7 was used, whereas for the *in vitro* experiments, a sample size of 3–5 biological replicates per group was used. The sample sizes in each group are indicated in the figures. The outliers were identified using Z scores (values >3 or < −3), and their origins were investigated. No outliers were excluded, as none were attributed to measurement errors. Statistical significance was set at a *P* value of less than 0.05.

## Results

### Effects of ghrelin treatment on body weight and blood glucose levels in control and diabetic rats

**Figure 1:**
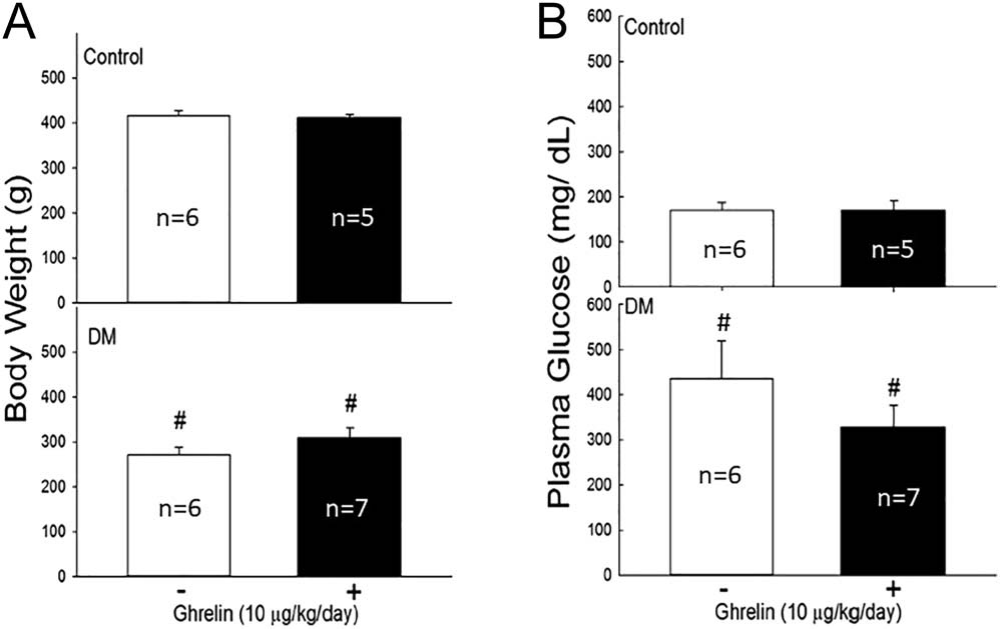
Effects of ghrelin on body weight and plasma glucose in rats. Ghrelin (10 μg/kg) was administered once daily for 7 days to both control (normal) and diabetic rats. Body weights and plasma glucose levels were measured. Each value represents the mean ± SEM. ^#^*P* < 0.05, compared with the control (DM effect).

As shown in Fig. 1A, the body weights of the diabetic rats significantly decreased after the induction of diabetes (*P* < 0.05) but did not significantly increase following ghrelin treatment.

Figure 1B shows that the blood glucose levels in the diabetic rats consistently remained within the diabetic range, which was defined as plasma glucose levels exceeding 275 mg/dL. Although ghrelin treatment reduced blood glucose levels in the diabetic rats, this reduction was not significant. These findings indicate that ghrelin treatment did not significantly alter hyperglycemia in STZ-induced diabetic rats over the course of 7 days.

### Effects of ghrelin treatment on testicular weight and morphology, as well as plasma testosterone, in control and diabetic rats

**Figure 2:**
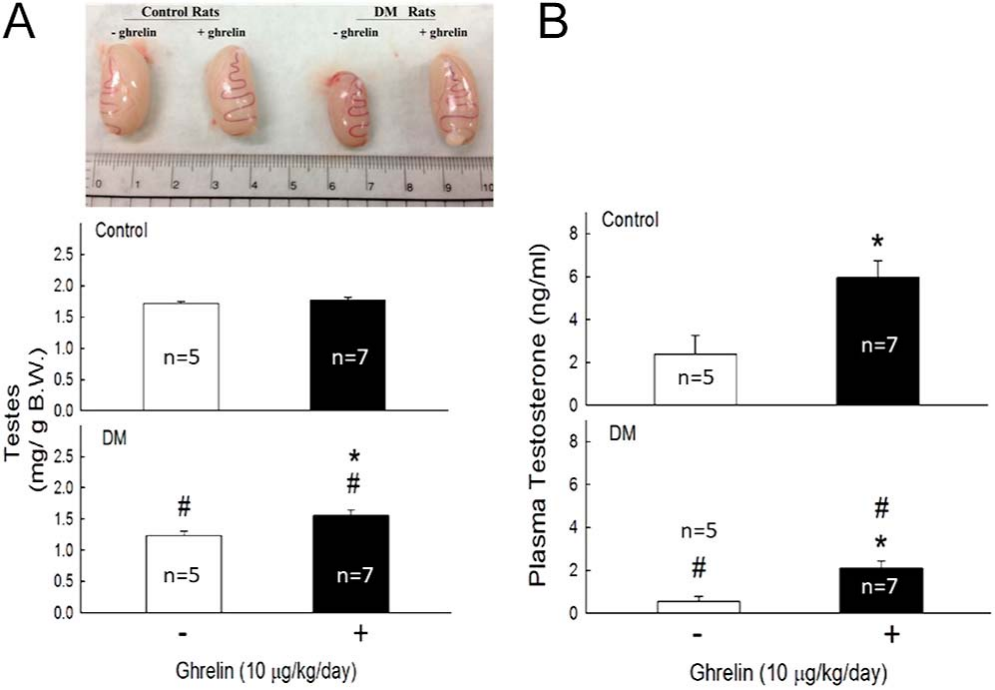
Effects of ghrelin on the weight of testes and plasma testosterone in control and diabetic rats. Ghrelin (10 μg/kg) was administered once daily for 7 days to both control (normal) and diabetic rats. The weight of the testes and the levels of plasma testosterone were measured. Each value represents the mean ± SEM. ^#^*P* < 0.05, compared with the control (DM effect); *P < 0.05, compared with the group treated without ghrelin (ghrelin effect).

As shown in Fig. 2A, there was a significant decrease in the testicular weights of the diabetic rats (*P* < 0.05). However, after ghrelin treatment was administered, the testicular weights of these diabetic rats significantly increased (*P* < 0.05). In contrast, ghrelin treatment exerted no effect on testicular weights in the control group. In addition, as shown in Fig. 2A, the testes appeared to be smaller in the diabetic rats.

Figure 2B shows a significant increase in plasma testosterone levels (*P* < 0.05) following ghrelin treatment compared with those rats in the control group. In the diabetic rats, plasma testosterone levels were significantly lower (Fig. 2B, *P* < 0.05) than those in the control group. However, after ghrelin treatment, these levels significantly increased (*P* < 0.05) and approached the levels of the control group. These findings indicate that ghrelin treatment can effectively increase plasma testosterone levels in both normal and diabetic rats.

### Effects of ghrelin *in vivo* on the basal and evoked testosterone secretion of Leydig cells *in vitro*, as induced by hCG, 8-Br-cAMP, and forskolin, in control and diabetic rats

The effects of ghrelin treatment on testosterone secretion were previously documented (see Fig. 2B); however, it was unclear whether this increase was due to increased testosterone production in Leydig cells or due to elevated testosterone levels in the body triggered by LH. To further investigate this phenomenon, Leydig cells from rats were isolated and treated with hCG to assess their responsiveness to LH.

**Figure 3:**
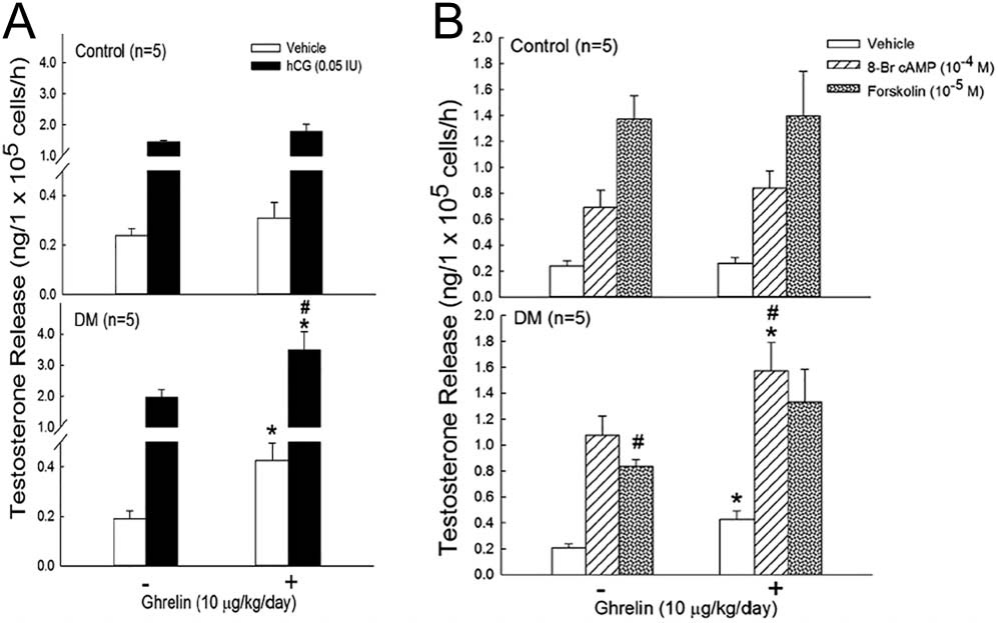
Effects of ghrelin on basal secretion and hCG-, 8-Br-cAMP-, or forskolin-induced testosterone secretion from Leydig cells in control or diabetic rats. Ghrelin (10 μg/kg) was administered once daily for 7 days to both control (normal) and diabetic rats. Leydig cells were isolated from the testes of both control and diabetic rats. Leydig cells were treated with or without hCG (0.05 IU), 8-Br-cAMP (10^−4^ M), or forskolin (10^−5^ M) in control and diabetic rats. The levels of testosterone in the media were measured. Each value represents the mean ± SEM. ^#^*P* < 0.05, compared with the control (DM effect); *P < 0.05, compared with the group treated without ghrelin (ghrelin effect).

As shown in Fig. 3A, neither the basal secretion nor the hCG-induced secretion of testosterone from Leydig cells differed from those of the control group after ghrelin treatment. However, in ghrelin-treated diabetic rats, the basal and hCG-induced secretions of testosterone from Leydig cells were significantly greater (Fig. 3A, *P* < 0.05) compared to diabetic rats that did not receive ghrelin treatment. In addition, the testosterone response to hCG in these ghrelin-treated diabetic rats was notably greater (*P* < 0.05) than that in normal rats treated with ghrelin.

Due to the fact that ghrelin was observed to increase testosterone secretion in the Leydig cells of diabetic rats (as illustrated in Fig. 3A), we further explored the use of cAMP stimulants. Figure 3B shows that neither basal testosterone secretion nor cAMP stimulant-induced testosterone secretion from Leydig cells changed in normal rats following ghrelin treatment. In contrast, in Leydig cells isolated from the diabetic rats, those treated with 8-Br-cAMP demonstrated significantly greater testosterone secretion (*P* < 0.05) compared to the diabetic rats that did not receive ghrelin treatment.

### Effects of ghrelin on basal secretion and GnRH-induced LH secretion in the APs, as well as the protein expression of LHR and StAR in Leydig cells, of control and diabetic rats

Ghrelin treatment increased plasma testosterone levels in both control and diabetic rats (see Fig. 2B). However, the *in vitro* release of testosterone from Leydig cells in normal rats treated with ghrelin did not change. Ghrelin influences not only the testes but also the hypothalamus and the pituitary gland. To further investigate these phenomena, the secretion of LH in the AP tissues of the rats was measured.

**Figure 4:**
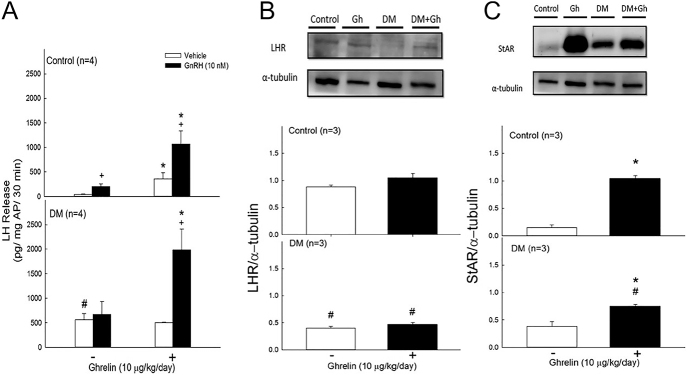
Effects of ghrelin on basal and GnRH-induced LH secretion in control and diabetic rats, as well as LHR and StAR protein expression in Leydig cells. Ghrelin (10 μg/kg) was administered once daily for 7 days to both control (normal) and diabetic rats. APs were cultured in medium supplemented with or without GnRH (10 nM) for 30 min in both control and diabetic rats. The LH concentration in the media was measured. Leydig cells were isolated from the testes of both control and diabetic rats. Cellular proteins were extracted from Leydig cells. The expression of the LHR protein, StAR protein, and α-tubulin was analyzed using Western blotting. Each value represents the mean ± SEM. ^#^*P* < 0.05, compared with the control (DM effect); *P < 0.05, compared with the group treated without ghrelin (ghrelin effect); ^+^*P* < 0.05, compared with the vehicle (GnRH effect).

As shown in Fig. 4A, ghrelin treatment significantly increased LH secretion in normal rats (*P* < 0.05). In the diabetic rats, the AP did not respond to GnRH stimulation. However, after ghrelin treatment, the response of LH secretion significantly increased (Fig. 4A, *P* < 0.05) and was even greater than that of the control group treated with ghrelin (*P* < 0.05).

Figure 4B shows that diabetes reduced LHR expression. Although ghrelin treatment increased LHR expression, this change was not statistically significant (Fig. 4B).

As shown in Fig. 4C, StAR expression in Leydig cells was observed to be increased after ghrelin treatment in both normal and diabetic rats (*P* < 0.05). Diabetes did not alter StAR expression; however, StAR expression was reduced in the diabetic rats following ghrelin administration (Fig. 4C, *P* < 0.05).

### Effects of ghrelin treatment on steroidogenesis in the Leydig cells of normal and diabetic rats

**Figure 5:**
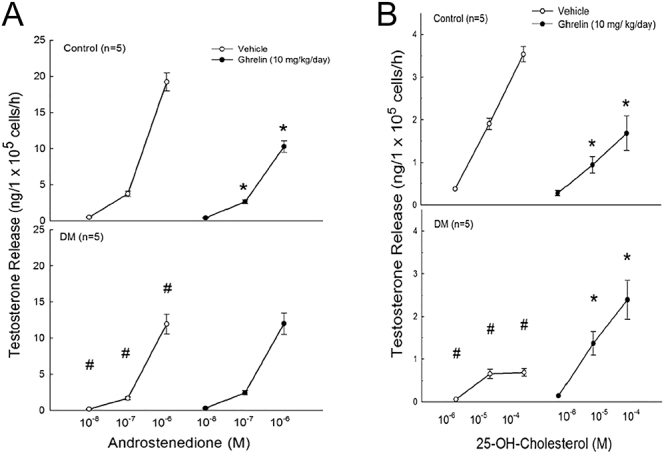
Effects of ghrelin treatment on steroidogenesis of Leydig cells in control rats and diabetic rats. Ghrelin (10 μg/kg) was administered once daily for 7 days to both control (normal) and diabetic rats. Leydig cells were isolated from the testes of both control and diabetic rats. Leydig cells were cultured in media with or without the precursors of testosterone biosynthesis, including 25-hydroxycholesterol (25-OH-C) or androstenedione (Δ4). The samples were collected, and the testosterone levels were analyzed. Each value represents the mean ± SEM. ^#^*P* < 0.05, compared with the control (DM effect); *P < 0.05, compared with the group treated without ghrelin (ghrelin effect).

The addition of androstenedione at concentrations of 10^−7^ M and 10^−6^ M to cultures of Leydig cells from normal rats resulted in a significant decrease in testosterone secretion following ghrelin administration (Fig. 5A, *P* < 0.05). Diabetes was associated with a decreased release of testosterone in response to androstenedione (*P* < 0.05). However, in ghrelin-treated diabetic rats, the presence of androstenedione did not alter testosterone secretion by Leydig cells (Fig. 5A). Compared with that in the diabetic group that did not receive ghrelin, testosterone production in the ghrelin-treated diabetic group in response to androstenedione remained unchanged (Fig. 5A). These results suggest that both diabetes and ghrelin treatment could reduce the activity of the enzyme 17β-HSD.

Figure 5B shows that the introduction of 25-OH-cholesterol at concentrations of 10^−5^ M and 10^−4^ M significantly decreased testosterone secretion in diabetic and ghrelin-treated rats (*P* < 0.05) when cultured with Leydig cells from normal rats. Conversely, in ghrelin-treated diabetic rats, Leydig cells stimulated with 10^−5^ M or 10^−4^ M 25-OH-cholesterol exhibited significantly increased testosterone secretion compared to diabetic rats that were not treated with ghrelin (Fig. 5B, *P* < 0.05). These findings suggest that ghrelin activated the enzymes P450scc and/or P450c17 in diabetic rats, thereby facilitating the conversion of 25-OH-cholesterol to pregnenolone and/or the conversion of pregnenolone to 17α-hydroxy-pregnenolone, which correspondingly increased testosterone secretion.

Moreover, the introduction of either androstenedione or 25-OH-cholesterol to diabetic rats resulted in decreased testosterone secretion in Leydig cells compared with that in normal rats (*P* < 0.05).

## Discussion

This study investigated the effects of ghrelin on testosterone production in normal and type 1 diabetic rats, with a focus on its mechanisms of production in Leydig cells and in the AP. Our findings demonstrated that ghrelin (10 μg/mL/kg, i.v.) enhances testosterone secretion *in vivo*, which is primarily achieved by increased LH secretion and the upregulation of steroidogenic acute regulatory (StAR) protein expression. *In vitro* experiments revealed distinct responses in diabetic rats compared to normal rats, thereby suggesting that diabetes modulates ghrelin efficacy via the cyclic adenosine monophosphate (cAMP) pathway and adenylate cyclase (AC) activity. Below, we discuss these findings in the context of diabetes-induced testicular dysfunction, analyze the key molecular mechanisms involved in the reported results, address unexpected results, and outline the limitations and future directions of the study.

### Diabetes-induced testicular dysfunction

Diabetes significantly impairs testicular function, as evidenced by reduced testicular weight, decreased testosterone secretion, and weakened LHR expression in Leydig cells. Our *in vivo* results confirmed that plasma testosterone levels are lower in diabetic rats than in control rats, which is consistent with prior reports of diabetes-related glandular dysfunction due to reduced Leydig cell numbers (Paz & Homonnai 1979). However, *in vitro* basal testosterone secretion from isolated Leydig cells (standardized to 100,000 cells) was comparable between the diabetic and control groups. This discrepancy suggests that the observed *in vivo* testosterone reduction may be due to a reduced Leydig cell population, rather than to an intrinsic defect in individual cell function (Paz & Homonnai 1979). The reduced testicular weights observed in the diabetic rats further support this hypothesis, with the results aligning with the results of previous studies demonstrating testicular atrophy in diabetes (Ballester *et al.* 2004).

In addition, our data indicate that diabetes weakens AC activity, as demonstrated by lower testosterone secretion in response to forskolin stimulation in diabetic Leydig cells compared to control cells. This finding is consistent with the literature suggesting that diabetes impairs cAMP signaling, which is a critical pathway for steroidogenesis (De Vriese & Delporte 2008). Despite increased LH secretion being observed in diabetic rats (which likely represents a compensatory response via the HPG axis), LHR protein expression in Leydig cells was reduced. This paradox may reflect desensitization of the LHR due to chronic LH elevation, which is a phenomenon that has been reported in diabetic models (Fernandez-Fernandez *et al.* 2005). The lack of significant LHR upregulation following ghrelin treatment (despite a slight increase) suggests that the effects of ghrelin on testosterone production are primarily mediated through other mechanisms, such as StAR protein expression (rather than via LHR modulation).

### The role of ghrelin in testosterone production

Ghrelin significantly enhances *in vivo* testosterone secretion in both normal and diabetic rats, which is primarily achieved by increasing LH secretion from the AP. In diabetic rats, ghrelin treatment also augments basal and stimulated (via hCG and 8-Br-cAMP) testosterone secretion *in vitro*, thus suggesting an increased sensitivity to ghrelin under diabetic conditions. Notably, the effect of ghrelin on LH secretion was more pronounced in diabetic rats than in control rats, where it had no significant effect on basal testosterone secretion. This differential response may be due to altered HPG axis regulation in diabetes, wherein low testosterone levels trigger compensatory LH secretion (Marques *et al.* 2000), which leads to the AP being more responsive to ghrelin. Previous studies have demonstrated that ghrelin modulates gonadotropin release in a dose-dependent manner, particularly under conditions of hormonal imbalance (Martini *et al.* 2006).

From an *in vitro* setting, ghrelin increased testosterone secretion in diabetic Leydig cells stimulated with 8-Br-cAMP (*P* < 0.05) but not in control cells, thereby indicating that ghrelin acts via the cAMP pathway in diabetic conditions. This observation aligns with previous reports showing that ghrelin activates downstream physiological responses via cAMP signaling (Camina *et al.* 2003). However, ghrelin exerted no effect on testosterone secretion when Leydig cells were stimulated with hCG, 8-Br-cAMP, or forskolin in controls, suggesting that normal Leydig cells may exhibit a saturated cAMP response, thereby limiting ghrelin’s efficacy. The reduced response to forskolin in diabetic rats further supports the notion that diabetes impairs AC activity, which ghrelin partially counteracts via cAMP-mediated pathways (De Vriese & Delporte 2008).

### Molecular mechanisms: StAR protein and steroidogenesis

The upregulation of StAR protein expression is a key mechanism underlying the ability of ghrelin to increase testosterone production. StAR facilitates cholesterol transport into the mitochondria, which represents the rate-limiting step in steroidogenesis (Strauss *et al.* 1999). In diabetic rats, ghrelin treatment significantly increased StAR expression, which was correlated with increased testosterone secretion in response to LH. These findings suggest that ghrelin sensitizes Leydig cells to LH by increasing cholesterol availability for steroid hormone synthesis. The biological implications of StAR upregulation are profound, as this protein directly influences the capacity for testosterone production and may potentially counteract diabetes-induced steroidogenic deficits (Miller & Bose 2011).

To further explore the effects of ghrelin on steroidogenesis, we examined the impacts of various steroidogenic intermediates (such as 25-OH-cholesterol and androstenedione). Ghrelin did not increase the conversion of androstenedione to testosterone, thus indicating that it does not activate 17β-hydroxysteroid dehydrogenase (17β-HSD), which is the enzyme responsible for this step (Labrie *et al.* 1997). However, when 25-OH-cholesterol was used as a precursor, ghrelin significantly increased testosterone secretion in diabetic Leydig cells, thereby suggesting that ghrelin enhances the activity of upstream enzymes, such as cytochrome P450 side-chain cleavage enzyme (P450scc) or P450c17. These enzymes catalyze the conversion of cholesterol to pregnenolone and subsequent intermediates (Payne & Hales 2004). This finding highlights ghrelin’s role in modulating early steroidogenic steps, particularly in diabetic conditions, wherein AC activity is compromised.

### Comparison with other protective agents

Recent studies have explored the effects of other agents, such as chrysin, safranal, and *Fumaria parviflora*, regarding their protective effects on testicular function. Chrysin mitigates testicular torsion/detorsion (TD)-induced injury by reducing oxidative stress (such as by reducing malondialdehyde (MDA) levels) and enhancing antioxidant defenses (including superoxide dismutase (SOD) and glutathione peroxidase (GPx)) (Abadi *et al.* 2023). It also improves sperm parameters, normalizes hormonal profiles (such as follicle-stimulating hormone (FSH), LH, and testosterone levels), and modulates apoptosis by decreasing Bax expression and increasing Bcl-2 expression (Abadi *et al.* 2023). In contrast, safranal primarily acts through antioxidant pathways, whereas *Fumaria parviflora* exerts effects via hormonal regulation in varicocele models (Dolatkhah *et al.* 2022, Ebrahimi *et al.* 2023). Unlike these agents, the effects of ghrelin reported in our study are mediated via LH secretion and StAR upregulation, with no direct evidence of antioxidant activity. These comparisons underscore the diverse mechanisms underlying testicular protection, which position ghrelin as a potential modulator of steroidogenesis (rather than as a broad-spectrum antioxidant).

### Critical evaluation of unexpected findings

Several unexpected findings warrant discussion. First, ghrelin increased LH secretion in diabetic rats but not in controls, despite similar *in vivo* testosterone responses being observed. This result may reflect a ceiling effect in normal rats, wherein basal testosterone levels are already optimal, thus limiting further LH-driven stimulation. In diabetic rats, hypersensitivity of the HPG axis to ghrelin may compensate for reduced testosterone, and this hypothesis has been supported by prior studies on gonadotropin regulation in diabetes (Martini *et al.* 2006). Second, ghrelin had limited effects on basal testosterone secretion in normal rats *in vitro*, and this effect is possibly due to sufficient endogenous cAMP levels, which ghrelin does not further increase in nonpathological states (Camina *et al.* 2003). Finally, the lack of significant LHR expression changes (despite increased LH secretion) in diabetic rats is surprising. This result may indicate that LHR signaling is saturated or that postreceptor defects (such as defects in AC activity) limit responsiveness, which is a phenomenon observed during chronic LH elevation (Fernandez-Fernandez *et al.* 2005). These findings highlight the context-dependent nature of the effects of ghrelin and the need for further mechanistic studies.

### Limitations and future directions

This study has several limitations. First, the small sample size may limit the statistical power for detecting subtle differences, such as the nonsignificant increase in LHR expression. Second, this study used a single ghrelin dose (10 μg/mL/kg), thus precluding dose–response analyses that could optimize therapeutic efficacy. Third, potential side effects of ghrelin (such as its impacts on appetite or growth hormone secretion) were not evaluated, which could confound its therapeutic application (Ebrahimi *et al.* 2023). Fourth, the study did not directly measure circulating LH or gonadotropin-releasing hormone (GnRH) levels; instead, it relied on inferred LH changes based on AP culture data, which limits the precision of our conclusions regarding HPG axis dynamics. Fifth, the absence of fertility endpoints (such as sperm count or motility) restricts our ability to assess the impact of ghrelin on reproductive outcomes (Agarwal *et al.* 2018). Finally, the utilized rat model may not fully recapitulate the condition of human type 1 diabetes, thereby necessitating validation in human-relevant systems.

Future studies should address these gaps via the following steps.

- Dose–response experiments should be conducted to determine the optimal ghrelin concentration for testosterone restoration.
- The effects of long-term ghrelin treatment on testicular function and systemic hormonal balance should be investigated.
- The side effect profile of ghrelin should be explored to assess its safety for clinical use.
- Circulating LH and GnRH levels should be measured to confirm HPG axis modulation.
- Fertility endpoints, such as sperm count and motility, should be evaluated to assess the impact of ghrelin on reproductive function.
- Findings in human Leydig cell models or clinical trials should be validated to confirm translational relevance.

### Therapeutic implications

Our findings suggest that ghrelin enhances testosterone production in diabetic rats by modulating LH secretion, StAR expression, and early steroidogenic enzymes. However, these results are preliminary, and further research is needed to establish the therapeutic potential of ghrelin. Clinical translation will depend on addressing the limitations of this study and confirming effects in human models. Until such data are available, ghrelin should be considered an experimental agent (rather than a definitive therapy for diabetes-induced testicular dysfunction).

## Conclusion

**Figure 6:**
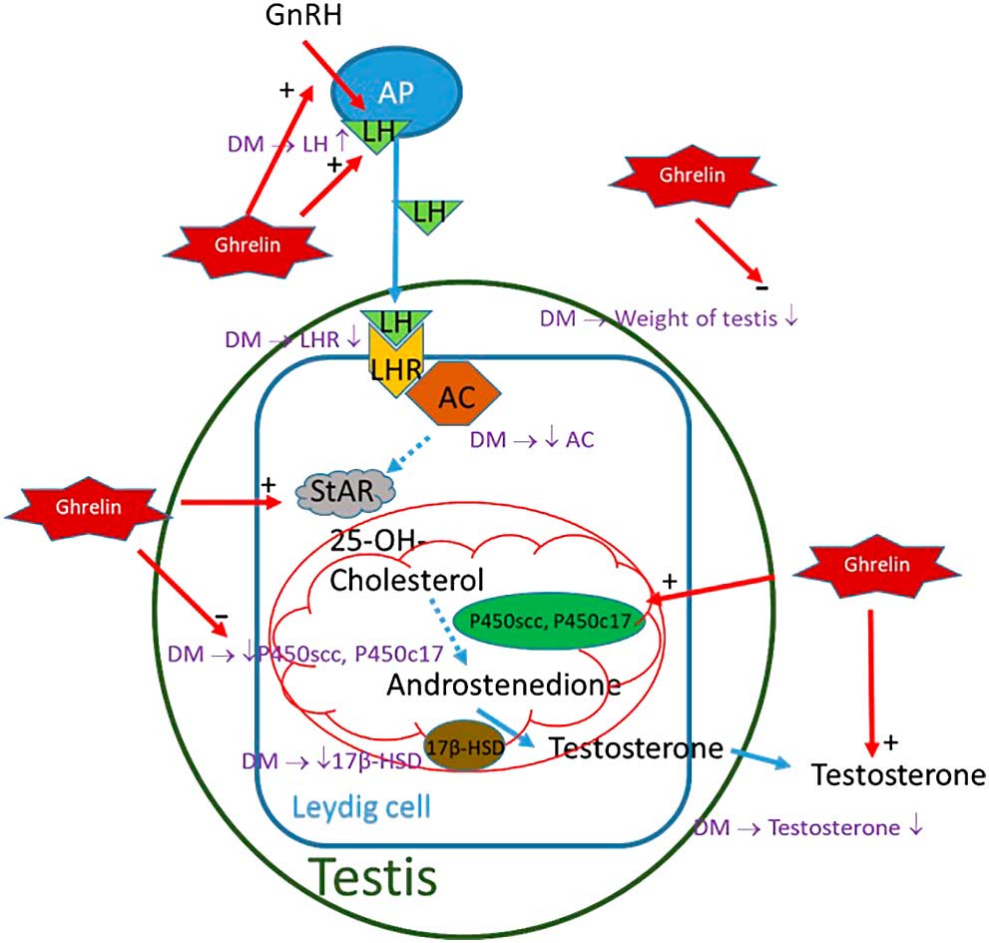
The mechanism of action of ghrelin on testosterone secretion in DM rats. Ghrelin enhanced LH secretion from APs in normal rats, amplified GnRH-induced LH release from APs in both normal and DM rats, and increased StAR protein expression in both groups. Ghrelin may also increase the activities of the enzymes P450scc and P450c17 in DM rats, thereby ultimately leading to increased testosterone production in rat Leydig cells.

In summary, ghrelin promotes testosterone production in normal and diabetic rats by increasing LH secretion and StAR protein expression, with additional effects on P450scc or P450c17 activity in diabetic conditions being noted (Fig. 6). These effects are mediated through the cAMP pathway, which is particularly responsive in diabetic Leydig cells. Compared with the mechanisms of action of agents such as chrysin, the mechanism of action of ghrelin is distinct and involves steroidogenic regulation (rather than antioxidative protection). Despite promising results, the limitations of the study and the need for further research underscore the preliminary nature of the therapeutic potential of ghrelin. These findings contribute to our understanding of ghrelin’s role in testicular function and provide a foundation for future investigations into its clinical utility.

## Declaration of interest

The authors declare that there is no conflict of interest that could be perceived as prejudicing the impartiality of the work reported.

## Funding

The study was supported by grants (MOST 103-2320-B-039-034 MY2 and MOST 104-2320-B-182-012) from the Ministry of Science and Technology, Taiwan, Republic of China. 

## Author contribution statement

CCL interpreted the findings, wrote the manuscript, and offered the research funding. CHC conducted the experiments, analyzed the data, and contributed to the literature review and background research. JCC and CCL completed the manuscript revision together. PLY and PSW conceived and designed the research framework. In addition, PSW assisted in applying for and successfully securing funding for this study from government agencies. The discussion section was structured by PSW and CCL, who also contributed valuable insights into the conclusion.

## Generative AI and AI-assisted technologies in the writing process

While revising this manuscript, the authors used Copilot and Grammarly for LLM- or AI-assisted copy editing, including grammar, spelling, punctuation, readability, and style improvements. These tools were not used for autonomous content creation. All text was initially written by the authors, who reviewed and approved all edits. The authors take full responsibility for the final content.
